# Structure–Function Changes of the Porcine Distal Outflow Tract in Response to Nitric Oxide

**DOI:** 10.1167/iovs.18-24943

**Published:** 2018-10

**Authors:** Susannah Waxman, Chao Wang, Yalong Dang, Ying Hong, Hamed Esfandiari, Priyal Shah, Kira L. Lathrop, Ralitsa T. Loewen, Nils A. Loewen

**Affiliations:** 1 Department of Ophthalmology, University of Pittsburgh Medical Center, Pittsburgh, Pennsylvania, United States; 2 Department of Ophthalmology, Xiangya Hospital, Central South University, Changsha, Hunan, China; 3 The Third Xiangya Hospital of Central South University, Changsha, Hunan, China; 4 Department of Ophthalmology, Peking University Third Hospital, Beijing, China

**Keywords:** distal outflow tract, SD-OCT, nitric oxide, aqueous flow, collector channels

## Abstract

**Purpose:**

To correlate outflow function and outflow tract vessel diameter changes induced by nitric oxide (NO).

**Methods:**

In a porcine anterior segment perfusion model, the effects of a nitric oxide donor (100 μM DETA-NO) on outflow facility were compared with controls (*n* = 8 per group) with trabecular meshwork (TM) and after circumferential ab interno trabeculectomy (AIT). Outflow structures were assessed with spectral-domain optical coherence tomography (SD-OCT) before and after NO, or an NO synthase inhibitor (100 μM L-NAME) and the vasoconstrictor, endothelin-1 (100 pg/mL ET-1). Scans were processed with a custom macroscript and aligned for automated reslicing and quantification of cross-sectional outflow tract areas (CSA).

**Results:**

The facility increased after DETA-NO (Δ of 0.189 ± 0.081 μL/min·mm Hg, *P* = 0.034) and AIT (Δ of 0.251 ± 0.094 μL/min·mm Hg, *P* = 0.009), respectively. Even after AIT, DETA-NO increased the facility by 61.5% (Δ of 0.190 ± 0.074 μL/min·mm Hg, *P* = 0.023) and CSA by 13.9% (*P* < 0.001). L-NAME + ET-1 decreased CSA by −8.6% (*P* < 0.001). NO increased the diameter of focal constrictions 5.0 ± 3.8-fold.

**Conclusions:**

NO can dilate vessels of the distal outflow tract and increase outflow facility in a TM-independent fashion. There are short, focally constricting vessel sections that display large diameter changes and may have a substantial impact on outflow.

Although the trabecular meshwork (TM) has long been considered the primary site of outflow resistance, its surgical removal or bypass does not lower the IOP to the predicted level of episcleral venous pressure.^1–3^ Recent laboratory studies showed that approximately 50% of outflow resistance is located further downstream.^4,5^ Our clinical studies of plasma-mediated ab interno trabeculectomy (AIT) show that the outflow resistance distal to the TM is higher in eyes with glaucoma.^6–9^ Only a small fraction of patients (∼0.3%) achieve the expected IOP.^9^ In fact, an empirical formula predicts that patients cannot achieve an IOP lower than 18.6 mm Hg without additional aqueous suppressants^10^ resulting in a failure rate of up to 30% within 12 months^6^ for a target below 12 mm Hg in moderate to severe glaucoma. The pre- and postoperative IOP in AIT are correlated,^6^ indicating an increased post-TM outflow resistance in eyes with a higher IOP. This suggests an incomplete understanding of outflow distal to the TM, and an avenue for new, targeted therapies. To explain the outflow resistance with the known numbers and diameters of distal outflow tract vessels,^11,12^ it has been speculated that not all outflow channels may be patent at the same time, or they could constrict and dilate.^5,13,14^ In theory, minute changes of small vessels, like collector channels (CC), could profoundly influence the facility. To generate the outflow resistance, they would have to have a diameter of only 20 μm, yet most have a diameter of approximately 50 μm or larger. A single vessel of this diameter could carry the entire flow,^5^ a principle applied to ab interno microgel stents to allow for a slow and safe aqueous humor drainage from the anterior chamber to the subconjunctival space.^15^ CC diameter changes from IOP variations^16^ and with the cardiac pulse wave^17^ do not explain the remaining distal outflow resistance, nor do valves at the orifices of collector channels^18^ as their removal by deep sclerotomy fails to reduce IOP further.^19,20^

NO increases trabecular outflow by acting on the guanylyl cyclase pathway.^21^ Muenster et al.^21^ showed that lambs exposed to topical NO had a lower IOP while mice breathing air containing 40 ppm NO had a lower IOP and an improved outflow facility. NO also dilates vessels by relaxing their smooth muscles^22^ via a protein kinase-dependent activation of K channels.^23^ Here, we hypothesized that it could increase the outflow facility in a TM-independent manner by dilating aqueous outflow tract vessels. We investigated its effect with and without circumferential AIT, developed an automated, quantitative, and live analysis of the cross-sectional area (CSA) of intrascleral outflow vessels, and searched for evidence of focal dilation and constriction that may show a reactive outflow regulatory mechanism.

## Materials and Methods

### Study Design

This study was designed as described in the following and detailed below. In experiment 1, eyes with intact TM were perfused at 4 μL/min. In group NO, media was supplemented with DETA-NO while controls (C) went through the same steps but with standard media (NO: *n* = 8, C: *n* = 8). In a pilot study used to establish suitability of 360° AIT as a method to remove TM and increase facility in porcine eyes, eight eyes were perfused at 6 μL/min to achieve a stable baseline prior to surgery, TM was removed circumferentially, and eyes were cultured for an additional 4 days. In experiment 2, the TM was removed over 360° by AIT. When pilot experiments in healthy eyes demonstrated a very low IOP, we increased the perfusion rate slightly to 6 μL/min. This increases the baseline IOP and makes the NO-induced IOP reduction easier to detect, thereby improving the power of statistical tests. Eyes in AIT-NO (*n* = 8) received DETA-NO supplemented media while controls (AIT-C, *n* = 8) went through the same steps but were perfused with conventional media. In experiment 3, outflow tract vessel dilation was quantified using wide-spectrum spectral-domain optical coherence tomography (SD-OCT) and received either NO-supplemented perfusion media (NO, *n* = 3) or L-NAME (N(omega)-nitro-L-arginine methyl ester) with ET-1-supplemented media (L-NAME + ET-1: *n* = 3).

### Anterior Segment Perfusion Culture

Porcine eyes were acquired on the day of experiments from a local abattoir (Thoma Meat Market, Saxonburg, Pittsburgh, PA, USA) and prepared for perfusion culture within 2 hours of euthanization as detailed previously.^24–27^ In brief, extraocular tissues and conjunctiva were trimmed away from globes. In an aseptic hood, globe exteriors were decontaminated via submersion in ophthalmic 5% betadine solution (NC9771653, Fisher Scientific, Waltham, MA, USA) for 2 minutes and rinsed three times with PBS (14080-055; Fisher Scientific). After hemisecting them, the choroid, iris, and ciliary body were carefully removed. The anterior segments were mounted on custom perfusion dishes, anterior chambers were filled with perfusion media (Dulbecco's modified Eagle medium (DMEM; sh30284.02, Fisher Scientific), 1% FBS (10082-147; Fisher Scientific), 1% antibiotic, antimycotic (15240-062; Fisher Scientific), and perfused with a microinfusion pump (70-3007; Harvard Apparatus, Holliston, MA, USA). In experiments 1 and 2, the eyes were cultured at 37°C with 5% atmospheric CO_2_ and perfused for more than 48 hours to acclimate and establish a stable baseline as done previously.^28^ A humidity pan was not used. IOPs were measured at 2-minute intervals with pressure transducers (Deltran II: DPT-200; Utah Medical Products, Midvale, UT, USA), recorded (FE224, PL3508/P, MLA1052; ADInstruments, Sydney, Australia), and analyzed (LabChart 7; ADInstruments). Cultures treated with NO received media supplemented with 100 μM diethylenetriamine nitric oxide adduct (DETA-NO, D185; Sigma-Aldrich Corp., St. Louis, MO, USA), an NO donor, prepared from powder on the same day of experiments. Due to a 57-hour half-life of DETA-NO in solution at room temperature and a 23-hour half-life at 37°C,^29^ fresh media was exchanged in syringes every 48 hours. Eight eyes were randomly assigned to treatment groups and perfused in parallel for approximately 1 week.

### Volumetric Wide-Spectrum Spectral-Domain Optical Coherence Tomography

We were unable to integrate the imaging system into a 37°C and 5% atmospheric CO_2_ environment due to its fairly large size, and devised a third, separate experiment to evaluate outflow tract structures. Segments were mounted on perfusion dishes, gravity perfused for 30 minutes at 15 mm Hg to acclimate eyes and establish a stable baseline, as done previously,^24,30–32^ and kept at room temperature throughout the experiments. Gravity perfusion was used for these experiments to allow for a rapid, yet gentle, pressurization and fluid exchange. Perfusion dishes were affixed to the center of a rotatable stage (XYR1; ThorLabs, Newton, NJ, USA; Supplementary Fig. S1) and placed under a 10-mm telecentric lens attached to the sample arm of a SD-OCT (Envisu R2210, Leica [Bioptigen], Morrisville, NC, USA). The sample arm was held in place with the adjustable carriage for a dissecting microscope, positioned so that the scanning beam was oriented perpendicularly to the limbus, and adjusted to focus on the tissue. The limbus was observed in the software's scan mode (InVivoVue; Bioptigen). The stage was rotated to locate a region in each eye where volumetric scans of at least 60° of consecutive limbus could be visualized. This included a small amount of cornea as a location reference, the distal sclera, and the signal voids of its corresponding vasculature in each scan. Scanned areas were 6-mm long (parallel to the limbus), 4-mm wide (cornea to distal sclera), and 1.6-mm deep. Due to the teardrop shape of the porcine cornea, the eye could not be rotated 360° around a stationary axis while viewing the limbus in a 4-mm wide window. Eyes were rotated 20° between scans. Between three and five baseline scans were obtained for each eye, depending on visualization. All scans were captured with enhanced-depth imaging. After baseline scans, perfusion media was supplemented with either 100 μM DETA-NO (*n* = 3) or with 100 pg/mL ET-1 and 100 μM L-NAME (*n* = 3) and an anterior chamber exchange was performed. After 40 minutes of gravity-based perfusion, scans were taken with the identical parameters and in the same regions.

### Image Processing

SD-OCT images were processed in ImageJ sotware^33^ (version 1.50i; http://imagej.nih.gov/ij/; provided in the public domain by the National Institutes of Health, Bethesda, MD, USA) and Amira Aviso (version 9.1, FEI; ThermoScientific) to remove noise, align pre- and posttreatment outflow tract signal voids in a three-dimensional (3D) space, and to allow automated, quantitative measurement of CSA. As detailed in Supplementary Data S1, SD-OCT files were converted to 8-bit TIFF stacks in ImageJ and cleaned with a custom ImageJ macroscript to denoise and extract signal voids from stacks. Cleaned and minimally processed stacks from both pre- and posttreatment scans of the same region were imported into Amira for 3D alignment and automated quantification of CSA.

### Statistical Analysis

In the pilot study, postsurgical data were compared with baseline with a Student's *t*-test in PASW 18.0 software (SPSS, Inc., Chicago, IL, USA). In experiments 1 and 2, facility changes were analyzed using a mixed-effect model^34^ in R.^35^ Averages were derived from 3-hour periods. Models were fitted using the lmer function in the lme4 package^36^ with fixed effects for treatment and random effects for subject and time point. All values were reported as the mean ± SEM unless otherwise stated to show the uncertainty around the estimate of the mean measurement.^37^ Small refill volume differences after media exchange in combination with a single pump rack that pushes up to eight syringes caused a higher range of IOPs during the first 6 hours in pilot experiments. IOP data recorded during these 6 hours were excluded from the analysis. Data are graphically presented as the mean facility change (Δfacility) over time. A *P* value less than or equal to 0.05 was considered statistically significant. In experiment 3, total values of pre- and posttreatment CSAs were compared by paired *t*-test.

## Results

### Outflow Facility Increase by NO Before and After TM Ablation

**Figure 1:**
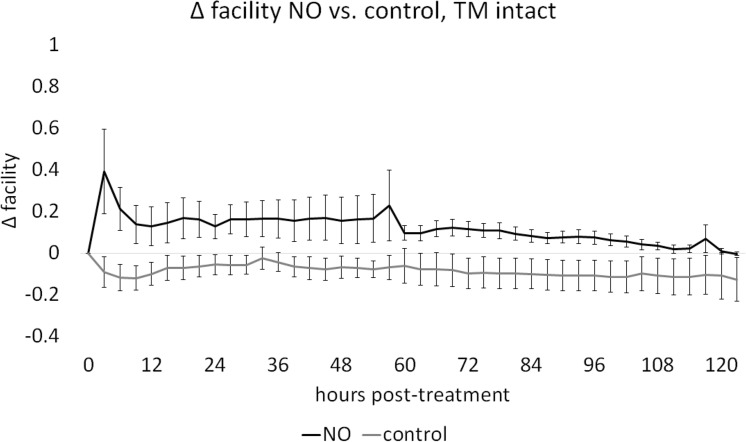
Effect of NO on facility in anterior segment cultures with intact TM. Outflow facility in NO remained above that of C throughout the experiment. Change in facility for C (−0.087 ± 0.010 μL/min·mm Hg) was compared with NO (0.113 ± 0.011 μL/min·mm Hg) with a mixed-effects model and showed an NO-mediated facility increase of 0.189 ± 0.081 μL/min·mm Hg (P = 0.034, n: NO = 8, C = 8).

**Figure 2:**
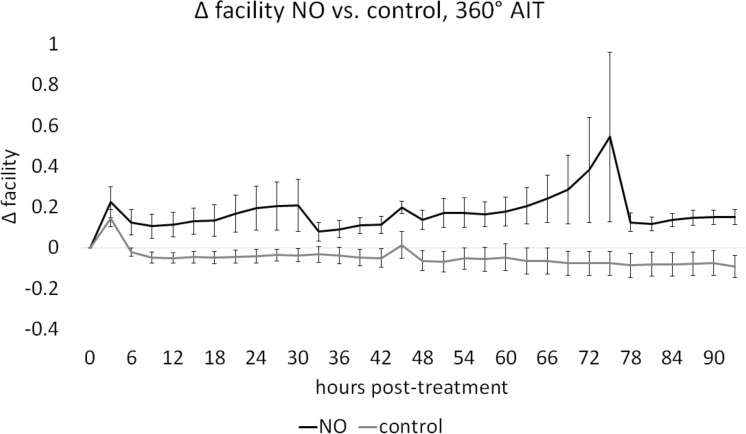
Effect of NO on facility in anterior segment cultures after TM ablation. Outflow facility in AIT-NO remained above that of AIT-C throughout the experiment. Posttreatment change in facility for AIT-NO (0.178 ± 0.021 μL/min·mm Hg) was compared with AIT-C (−0.054 ± 0.009 μL/min·mm Hg) with a mixed-effects model and indicated an NO-mediated facility increase of 0.190 ± 0.074 μL/min·mm Hg (P = 0.023, n: AIT-NO = 7, AIT-C = 8).

In cultures with intact TM, baseline facilities in NO and C were not different (*P* = 0.80). Posttreatment facility of C, with a mean decrease of 0.087 ± 0.010 μL/min·mm Hg, was compared with NO, with a mean increase of 0.113 ± 0.011 μL/min·mm Hg, through a mixed-effects model, indicating a significant NO-mediated facility increase of 0.189 ± 0.081 μL/min·mm Hg (46.83%, *P* = 0.034, NO: *n* = 8, C: *n* = 8, Fig. 1). Circumferential ablation of TM was achieved and induced a significant facility increase (0.251 ± 0.160, 59.2%) from baseline (*P* < 0.001). Eyes were perfused before and after AIT, in groups AIT-NO and AIT-C as in our prior experiments.^38–42^ One eye had to be excluded due to contamination (N: AIT-NO = 7, AIT-C = 8). Baseline facility measures in AIT-NO and AIT-C were not different (*P* = 0.37). Posttreatment facilities for AIT-C, with a mean change of −0.055 ± 0.009 μL/min·mm Hg, showed a slight and steady decrease throughout the experiment while facilities in AIT-NO, with a mean change of 0.179 ± 0.021 μL/min·mm Hg, were increased from baseline at all posttreatment time-points. After AIT, a mixed-effects model showed a significant NO-mediated facility increase of 0.190 ± 0.074 μL/min·mm Hg (61.49% *P* = 0.023, Fig. 2). The average baseline IOP in constant-rate infusion experiments was 15.32 ± 1.37, similar to the pressure used in gravity-based infusion experiments.

### Volumetric SD-OCT Scanning and Image Processing

**Figure 3:**
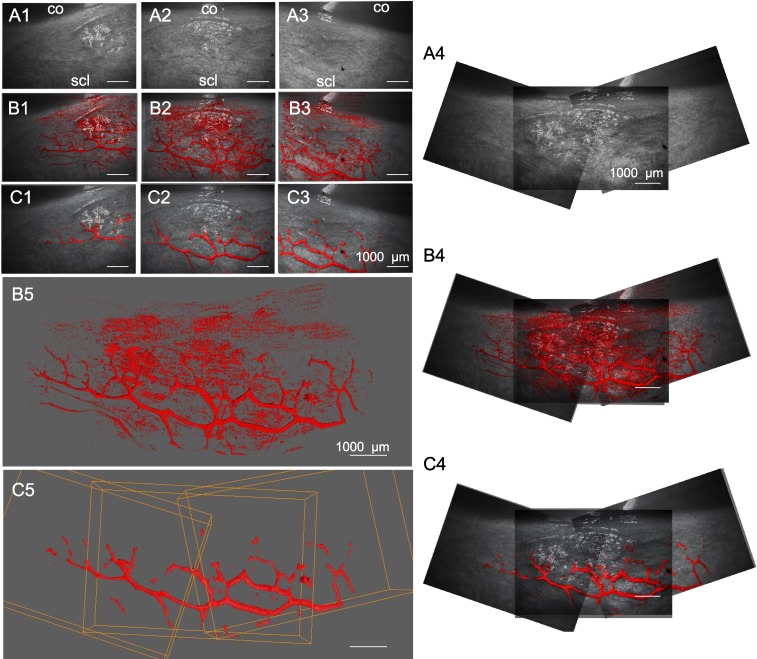
Rendering and alignment of adjacent outflow tract vessel volumes in 3D. Volume intensity projections (VIPs) (A1–3) of adjacent OCT scans with minimally processed (B1–3) and cleaned signal voids (C1–3) in overlay. (A4–C4) 2D alignment of images in (A–C), 1 to 3 fails to account for the curvature of the ocular surface, resulting in alignment artifacts. (B5) surfaces in (B1–3), and (C5) in (C1–3) are aligned in 3D space to neighboring scans without artifacts. Bounding box (orange) coordinates in (C5) were conferred to respective scans in (B5) to align them. co, cornea; scl, sclera.

Limbal outflow structures were visualized successfully. A contrast agent was not necessary. Previous experiments indicated a flow rate of 3.7 ± 1.6 μL/min at a constant gravity perfusion with a physiologic pressure of 15 mm Hg^43^ equivalent to 20.3-cm water column.^24^ Volumetric SD-OCT scans were captured at a minimum of three adjacent locations for each eye. A single scan required 21 seconds. ImageJ processing of SD-OCT volumes with the custom macroscript could isolate intrascleral signal voids within outflow tract vessels of the same regions shown in corresponding, unprocessed B-scans. Compared with previous grayscale inversion and background subtraction alone, there was reduced noise and minimal loss of signal. Scans could be manipulated in a virtual 3D space to view structures from all angles and showed good agreement with outflow tract vessels visible in confocal microscopy.^12^ Compared with minimally processed scans (Figs. 3B1–5), features of structures were well preserved in cleaned scans with sufficient noise reduction (Figs. 3C1–5).

### NO Induces Outflow Vessel Dilation and Releases Focal Restrictors

**Figure 4:**
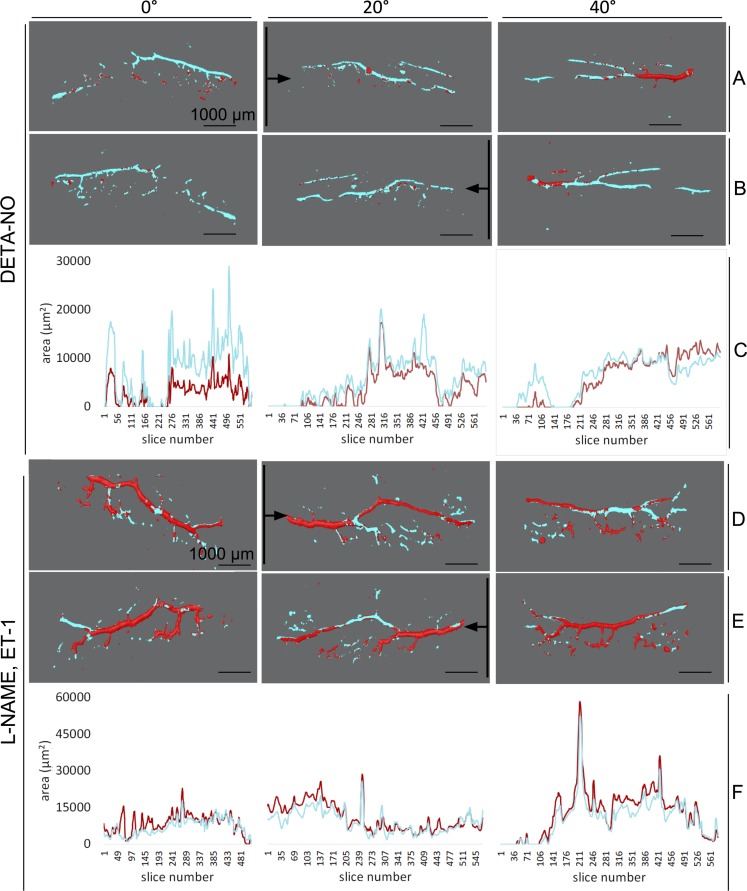
Quantification and of cross-sectional area pre- and posttreatment. Aligned and merged surface reconstructions taken at 0°, 20°, and 40° with pretreatment pseudocolored in red and posttreatment in cyan as viewed anteriorly (A, D) and posteriorly (B, E). Eyes treated with L-NAME + ET-1 showed a greater coverage with red throughout, indicating larger pretreatment than posttreatment intraluminal space (constriction), and DETA-NO–treated vessels show greater coverage with cyan, indicating a larger posttreatment than pretreatment intraluminal space (dilation). CSA of virtual sections, made along the plane indicated by the black lines and in the direction of the black arrows, was calculated in silico. CSA is plotted throughout the length each sample (C, F).

**Figure 5:**
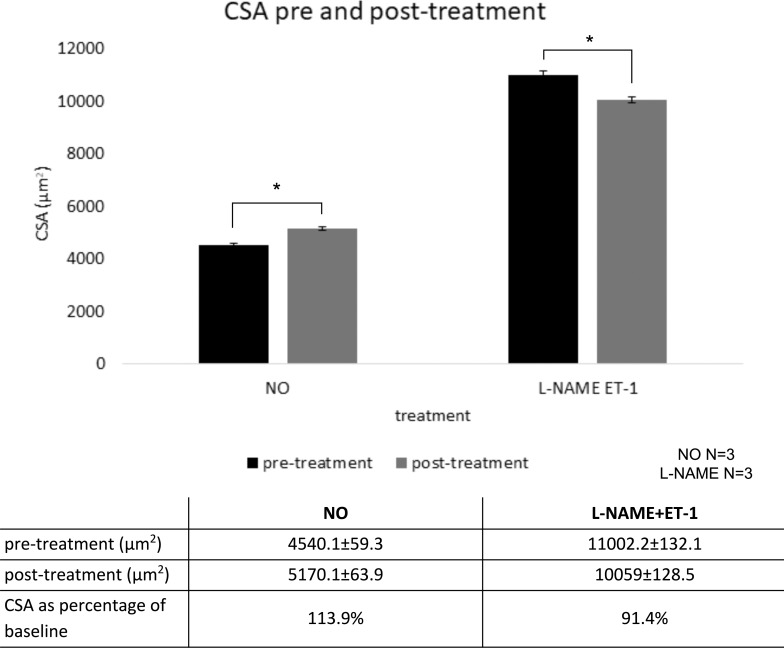
Cross-sectional area of outflow tract vessels pre- and posttreatment. N = 3 eyes per treatment group; 13,146 cross-sections were analyzed in NO and 10,580 in L-NAME + ET-1. *P ≤ 0.001, values reported as mean ± SEM.

CSA values for each 3D rendering could be automatically quantified and reported by the software along with the corresponding cross-section location. As seen in 3D renderings of scans from a representative DETA-NO–treated eye (Figs. 4A–C, 0°–40°), a larger wrapping with cyan in most locations corresponded with higher CSA values in the corresponding plots below (see Fig. 8C, 0°–40°). Conversely, in the 3D renderings of a representative L-NAME + ET-1–treated eye (Figs. 4C, 4D, 0°–40°), more extensive coverage of pretreatment (red) in most areas corresponded with higher overall CSA values in the corresponding plots below (Figs. 4D–F, 0°–40°). Posttreatment CSA was compared withs pretreatment baseline in of each three eyes treated with DETA-NO (22 scans total, 13,146 virtual sections) and three eyes treated with L-NAME + ET-1 (18 scans total, 10,580 virtual sections). DETA-NO increased CSA by an average of 13.9% and L-NAME + ET-1 decreased CSA by an average of 8.6% (Fig. 5, *P* < 0.001 for both groups). CSAs from each eye demonstrated the expected trend (Supplementary Table S2). CSA calculation according to the Amira output was verified in ImageJ for five slices, and CSA values corresponded precisely.

**Figure 6:**
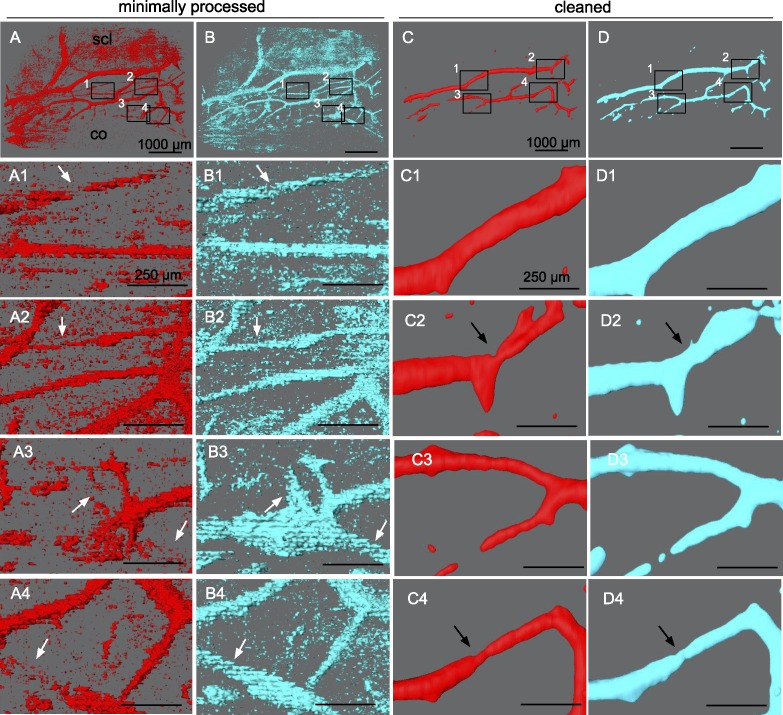
NO-induced focal dilation of outflow tract vessels. Outflow tract vessels pre- (A, C, red) and post-treatment (B, D, cyan) with DETA-NO vasodilator. Insets 1 to 4 in (A–D) show the location of magnified (A1–D4). Dilation of outflow tract vessels can be seen in minimally processed reconstructions of SD-OCT signal voids (A4–B4). Outflow tract vessels can be seen to open at previously closed locations (white arrows), dilate at focal sites of high response (black arrows), and throughout their lengths.

**Figure 7:**
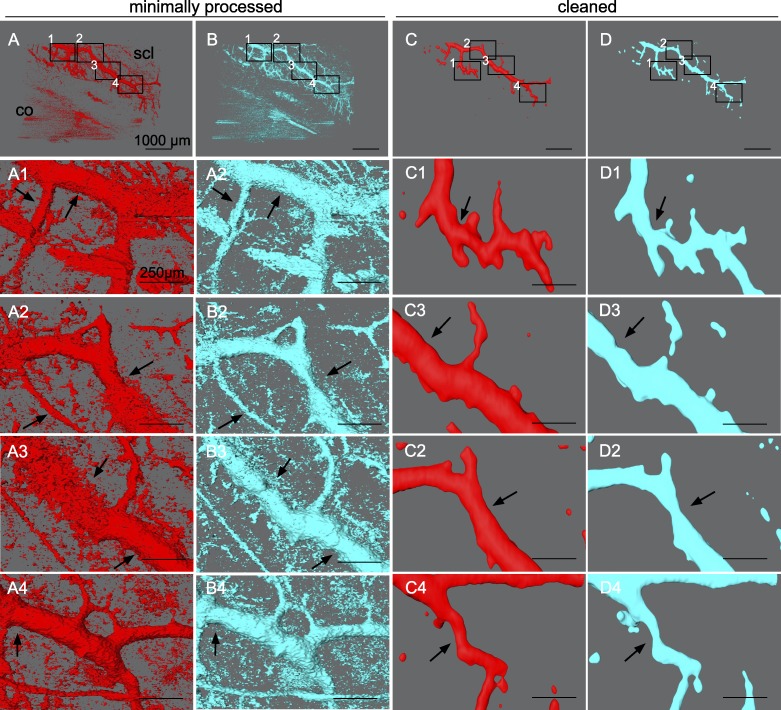
L-NAME + ET-1–induced focal constriction of outflow tract vessels. Outflow tract vessels pre- (red) and posttreatment (cyan) with L-NAME and ET-1 vasoconstrictors. Constriction of outflow tract vessels can be seen in projections of minimally processed scans (A–B4) as well in as cleaned scans used for quantitative slice-by-slice comparison (C–D4). Insets 1 to 4 in (A–D) indicate the location of the magnified (A1–D4). Outflow tract vessels can be seen to constrict or collapse at focal sites of high response (black arrows), and constrict throughout their lengths.

Besides quantitative evaluation, structural changes of outflow tract volume could be appreciated in 3D. In NO (Fig. 6), outflow tract vessels appeared to open closed locations (white arrows), release focal restrictors at sites of high response (black arrows), and dilate throughout. In contrast, outflow tract vessels in L-NAME + ET-1 (Fig. 7) constricted or collapsed at sites of high response (black arrows). Focal restrictors had a diameter increase to 502 ± 376% after NO (Fig. 6). Conversely, L-NAME + ET-1 caused a focal constriction to 53 ± 9% (Fig. 7). While cleaning removed visual noise at the expense of removing the signal from more delicate structures, minimally processed scans (Figs. 6, 7, A1–B4) showed 3D patterns similar to the cleaned data used for quantitative slice-by-slice analysis (Figs. 6, 7, C1–D4).

## Discussion

This study determined functional and structural effects of NO on the conventional outflow tract distal to the TM. We found that NO, a messenger and hypotensive compound, increased the facility even after circumferential AIT. Our work agrees with the recent McDonnell et al.^44^ recent study, which describes the ability of DETA-NO to acutely increase facility even after TM removal not only in porcine but also in human eyes. Here, we confirmed those findings and extended the analysis by showing a facility change for up to 5 days. We showed that vasodilation of outflow vessels can be directly observed by SD-OCT, including focal flow restrictors and vessels small enough to impact the facility, as theorized to exist.

Because unconjugated NO has a half-life of several seconds,^45^ we chose DETA-NO due to its relative stability compared with other NO donors with more rapid NO release. The hypotensive effects of DETA-NO have been established in both porcine and human anterior segment perfusion culture,^46,47^ including after TM removal.^44^ Because NO can compensate for ET-1 mediated vasoconstriction^48,49^ in healthy eyes, the NOS inhibitor L-NAME was used in conjunction with physiologic ET-1 levels to induce a maximally constricted phenotype as a positive control. NO supplementation was used in the absence of preconstricting agents, unlike in the work by McDonnell et al.,^44^ to determine its standalone physiologic effects relevant in future therapies. Pilot studies done on anterior segments perfused with drug-free media indicated no trend in perfusion-mediated changes of outflow tract caliber within the experimental timeframe for experiment 3 (Supplementary Fig. S2). Because of the short time from recovery to culture, we did not have to use a NO synthase (NOS) inhibitor or precontraction agent as may be necessary in human donor eyes with variable freshness to observe a hypotensive effect.^44^ Absent of a parallel gravity fluid reservoir for all eyes, our experimental setup required us to focus on constant-rate infusion data collected 6 hours after a media exchange. Our data spans several days and complements well the more acute outflow study by McDonnell et al.^44^ well, which does include the first 6 hours. TM ablation by AIT was performed over the entire circumference in this study to create direct access to the collector channels and eliminate TM-based outflow resistance.

Consistent with our prior experience,^24,26–28,41,50^ the perfused porcine anterior segments did not experience the amount of corneal edema reported with other culture techniques.^51^ With intact TM, NO increased the facility by approximately 45% in group NO compared with the control group, C. After 360° AIT in experiment 2, we found an NO-mediated facility difference of approximately 60% between AIT-NO and AIT-C, in line with the findings reported by McDonnell et al.^44^ This difference persisted throughout experiments 1 and 2 but declined toward the end of experiment 1, possibly due to a decreasing TM facility response to NO. The TM makes for a significant contribution to outflow resistance, but not in experiment 2 where the facility enhancement is TM independent. Trabectome-mediated TM ablation results in an approximately 300 μm wide excision down to the bare sclera^27,38–41,50,52^ where the opening of the collector channels are located without significant trauma or thermal injury. Like DETA-NO, cromakalim treatment of mouse eyes lowered episcleral venous pressures, and in TM-free human anterior-segment cultures, lowered IOP.^53^ These results suggest a target for pharmaceutical manipulation of post-trabecular outflow resistance that may be especially relevant after microincisional surgeries that remove or bypass the TM.

We examined the outflow tract vessels directly with SD-OCT, which allowed us to obtain full-thickness image stacks of the perilimbal sclera.^54–56^ We improved this method by automating segmentation and assembly for a faster and more objective process. It was possible to isolate signal voids and reconstruct them into a complex outflow tract vessel network without the need to fix and clear this tissue as we have done before,^12^ but at the cost of a lower resolution. While most features were preserved after processing scans, it is important to recall that any denoising can cause loss of details of the structure. Confocal microscopy can produce high-resolution volumes as well, but in order to reach comparable imaging depth, tissue must be fixed and cleared. Higher resolution imaging of living tissue can also be achieved with two-photon microscopy, but the imaging depth is more limited and could not be used here.^5^ Additionally, while both facility and structural information was collected in eyes used in separate experiments, these factors were not evaluated simultaneously in the same eye. This is a limitation of the current study.

Automated reconstruction of the outflow tract from SD-OCT signal voids allowed direct observation of vasomotion in vessels as large as 200 μm and as small as 20 μm in diameter. NO induced extensive vasodilation throughout the distal outflow tract while L-NAME + ET-1 caused substantial constriction. While these diameter changes alone might alter the outflow resistance, we observed many focal vessel segments that constrict and dilate significantly more. They may be the anatomic correlate for the rather dynamic outflow patterns observed in human patients and nonhuman primates.^58^ Smooth muscles surrounding collector channels,^57^ and NOS expression by SC and CC endothelium,^59,60^ suggest a site-specific capacity for distal outflow tract caliber regulation and segmental flow changes.^13,61^ Recently introduced NO-donating prostaglandin analogs^62,63^ may help address the lower levels of NO in eyes of POAG patients and an overlooked, posttrabecular cause of reduced facility.^64–66^

In conclusion, while the TM is recognized as a principal site of conventional outflow resistance and regulation, we show that NO increases the outflow facility for as long as 5 days, both with intact TM and after circumferential AIT. NO dilates distal outflow tract vessels while L-NAME + ET-1 constricts them. Five-fold focal caliber changes can be observed.

## Supplementary Material

Supplement 1
