# Supplementary material for: Structure–Function Changes of the Porcine Distal Outflow Tract in Response to Nitric Oxide
Source: Invest Ophthalmol Vis Sci. 2018 Oct;59(12):4886–95. doi: 10.1167/iovs.18-24943 (PMC6181305; doi:10.1167/iovs.18-24943)
Supplement: Supplement 1 [file iovs-59-11-46_s01.pdf]

Supplementary Figure 1: SD-OCT imaging setup

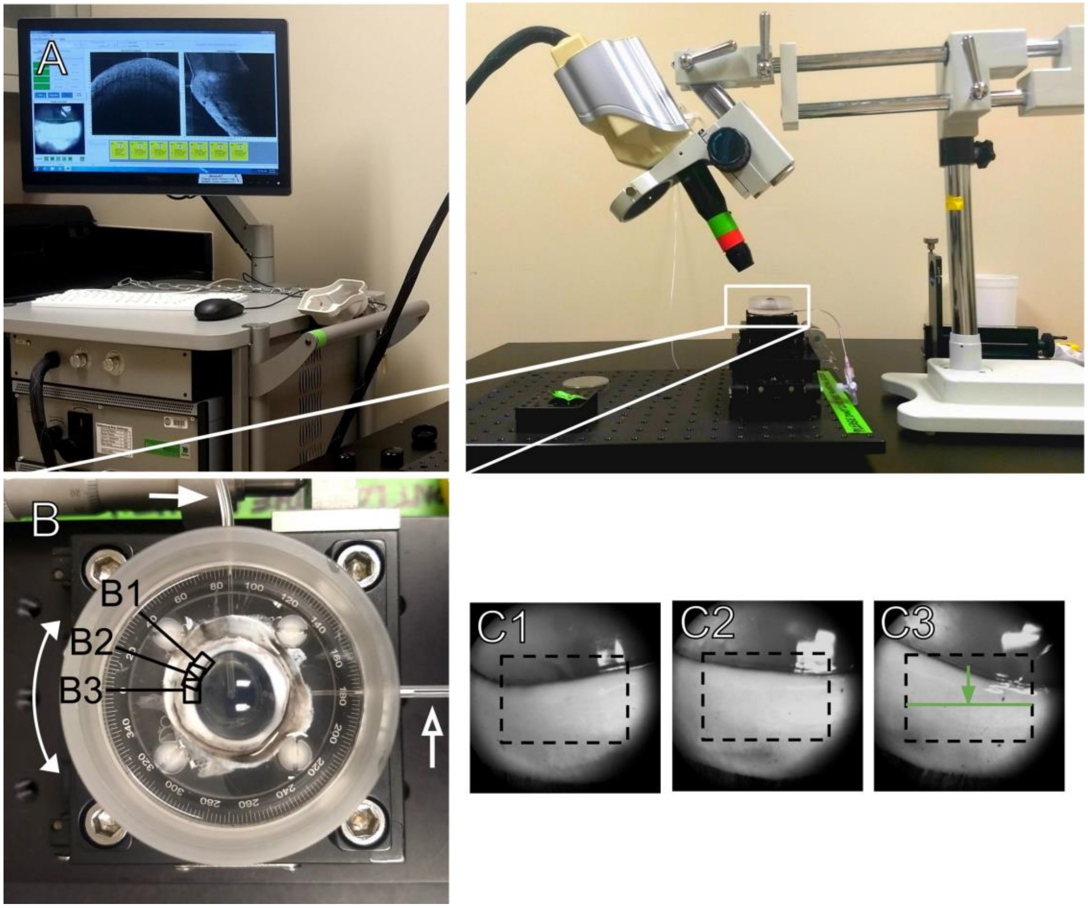

Supplementary Figure 1: SD-OCT imaging setup. A) Anterior segments were affixed to a rotatable stage (white box) under the scanning arm of an R2210 Envisu SD-OCT. Before image capture, limbal tissue was visualized in Bioptigen InvivoVue software through a 10 mm telecentric lens. B) Anterior segment in its perfusion dish, the infusion line (straight, closed arrow), the outlet (open arrow), direction of dish rotation (curved arrow) and the location of scans along the limbus (black boxes B1-B3). C1-C3) Representative sample areas corresponding with locations B1-B3, each 20° apart. The plane and direction of B-scan capture is indicated in green. Scans were acquired over a sample area 6.0 mm long, 4.0 mm wide, and 1.6 mm deep with 1000x600x1024 pixels.

Supplementary Figure 2: Drug-free vessel caliber control

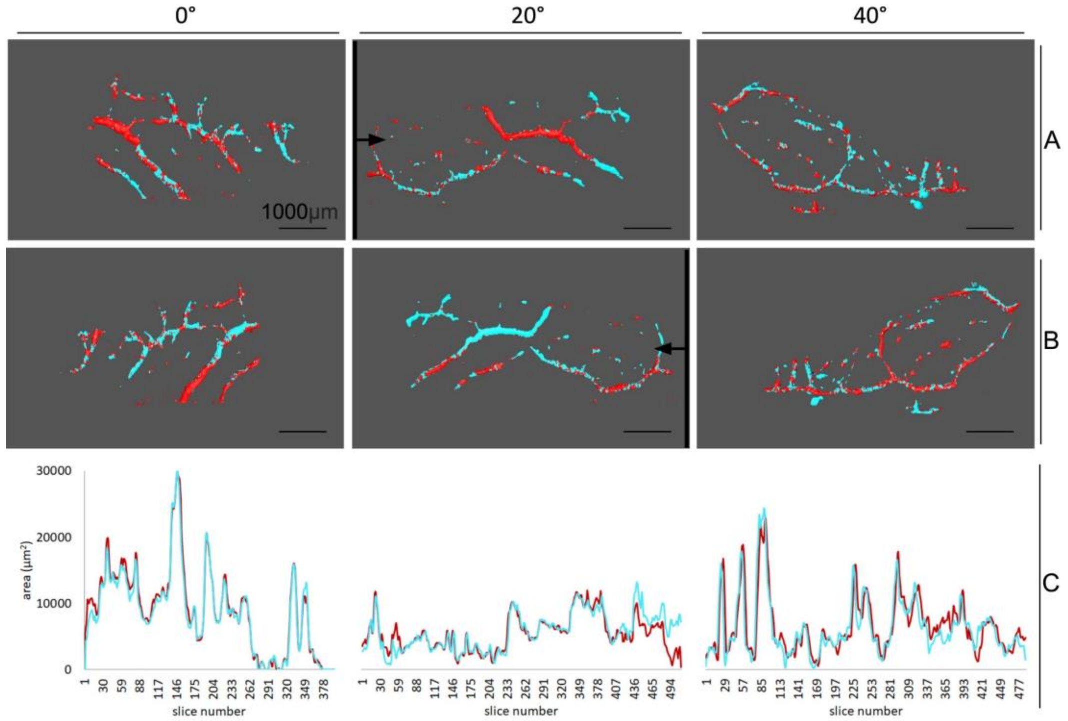

Supplementary Figure 2: Drug-free vessel caliber control

Aligned and merged surface reconstructions taken at 0, 20, and 40° with pre-treatment pseudocolored in red and post-treatment in cyan as viewed anteriorly (A) and posteriorly (B). Pilot anterior segments were perfused with drug-free control media for 40 minutes. Cross-sectional area (CSA) of virtual sections, made along the plane indicated by the black lines and in the direction of the black arrows, was calculated *in silico*. CSA is plotted throughout the length each sample (C). In these 2,868 virtual sections, with approximately equal regions of red and cyan throughout 3D reconstructions, and there was no consistent trend of increase or decrease in CSA from baseline throughout scans.

Supplementary Table 1: raw facility data

treatment	baseline facility	post-treatment facility
NO	0.404±0.106	0.516±0.023
C	0.445±0.103	0.358±0.009
AIT-NO	0.309±0.021	0.488±0.022
AIT-C	0.562±0.160	0.515±0.023

values reported as mean ± standard error

Supplementary Table 2: CSA pre-and post-treatment

sample ID	pre-treatment CSA (μm^2) ± SEM	post-treatment CSA (μm^2) ± SEM	Δ CSA (% change)
eye 1, DETA-NO	2959.2 ± 89.6	3346.0 ± 95.8	386.7 ± 44.9 (13.1)
eye 2, DETA-NO	3406.4 ± 117.1	3455.4 ± 124.5	49.0 ± 44.6 (1.4)
eye 3, DETA-NO	6157.6 ± 86.8	7277.6 ± 90.2	1120.0 ± 39.9 (18.2)
eye 4, L-NAME + ET-1	14818.2 ± 286.5	14399.6 ± 287.0	-418.6 ± 84.0 (-2.8)
eye 5, L-NAME + ET-1	10103.0 ± 173.4	8329.8 ± 142.5	-1773.2 ± 61.7 (-17.6)
eye 6, L-NAME + ET-1	8121.5 ± 179.2	7475.9 ± 174.4	-645.6 ± 54.2 (-7.9)

values reported as mean ± standard error
